# A mechanistic approach to studies of the possible digestion of retrograded starch by α-amylase revealed using a log of slope (LOS) plot

**DOI:** 10.1016/j.carbpol.2014.06.089

**Published:** 2014-11-26

**Authors:** Hamung Patel, Richard Day, Peter J. Butterworth, Peter R. Ellis

**Affiliations:** King's College London, School of Medicine, Diabetes and Nutritional Sciences Division, Biopolymers Group, Franklin-Wilkins Building, 150, Stamford Street, London SE1 9NH, UK

**Keywords:** Starch, α-Amylase, Log of slope plot, First-order kinetics, FTIR-ATR, Retrogradation

## Abstract

The rate and extent of digestibility of starch were analysed using the logarithm of the slope (LOS) method. Digestibility curves with α-amylase were obtained for starches in their native, gelatinised and 24 h retrograded form. A LOS plot of the digestibility curves was then constructed, which allowed the rate constant (*k*) and the concentration of the product at the end of the reaction (*C*∞) to be calculated. It also allowed the identification of rapid and slow phases in starch digestion. Upon gelatinisation, both *k* and *C*∞ increased with dramatic changes notably in *C*∞; however after starch samples had been stored for 24 h at room temperature, *k* was not affected but *C*∞ decreased. This suggests that retrograded starch is virtually inert to amylase action. Both *k* and *C*∞ were strongly related to the increase in degree of order of the α-glucan chains, monitored by FTIR-ATR spectroscopy, in retrograded starch.

## Highlights

• Digestion of native and processed starches can be fitted to the LOS plot model.
• RDS and SDS fractions identified for the digestion of different native starches.
• *C*∞ reduced for retrograded starches while *k* remained constant.
• Retrograded starch is completely inert to amylase attack.
• Ordered starch material was investigated by FTIR-ATR.

## 1 Introduction

Starch is the main source of digestible carbohydrate and it contributes significantly to the total energy intake of food in the human diet. Amylose and amylopectin are the two major α-glucan polymers of starch, with amylopectin having a higher average molecular weight and a more branched structure than amylose. Both contain glucose units joined by α 1–4 glycosidic bonds, which can be hydrolysed by α-amylase to produce oligosaccharides, especially maltose. The branches in amylopectin are formed through α 1–6 glycosidic bonds, which are linkages that are resistant to α-amylase action (Pérez & Bertoft, 2010). Oligosaccharides resulting from amylolysis are hydrolysed to glucose by the mucosal glucosidases (amyloglucosidase and sucrase–isomaltase) of the small intestine, absorbed by epithelial cells and eventually transferred to the peripheral blood circulation.

It is now widely documented that starch-rich food products with similar starch contents can produce different postprandial blood glucose and insulin responses in human subjects. Therefore there is considerable interest in understanding the basis for the observed differences in rates of intestinal starch digestion following a starch-rich meal. These differences have prompted studies of factors, such as the chemical and physical structure and properties of starch at the molecular and granular levels, that affect the rate and extent of α-amylase action on numerous native and hydrothermally processed starches (Butterworth et al., 2012, Warren et al., 2012, Warren et al., 2013).

Previous studies have involved *in vitro* starch digestion with pancreatic α-amylase to mimic *in vivo* amylolysis and predict the likely *in vivo* glycaemia resulting from α-amylase acting on starch-rich food materials. This allows foods to be classified on the basis of their potential glycaemic index. The *in vitro* approach to determining the rate of starch digestion has advantages over *in vivo* studies (animal and/or human subjects) in being considerably cheaper to perform and no ethical authorisation is required. In addition, the *in vitro* experiments are less time consuming (Butterworth et al., 2011, Butterworth et al., 2012, Mahasukhonthachat et al., 2010).

Englyst and Cummings (1987) classified starches into rapidly digested (RDS) and slowly digested (SDS) fractions based on digestibility data obtained from *in vitro* incubations with α-amylase. In this scheme, RDS is the fraction which is digested within the first 20 min of incubation with amylase and SDS is measured from the amount digested between 20 and 120 min. Undigested starch remaining from *in vitro* amylolysis is classified as resistant starch (RS), which in studies performed *in vivo* is known to escape digestion in the small intestine (Englyst & Cummings, 1987). This classification system is seriously flawed given that the amylolysis of cooked/processed starch materials is described by a pseudo-first-order kinetic process characterised by a single digestibility constant (Butterworth et al., 2011, Dhital et al., 2010, Goñi et al., 1997). First-order kinetics proves that any digestible material in processed starch, or starch-containing foods, has the same intrinsic reactivity with respect to amylase. The slowing of rate as the reaction proceeds is a natural consequence of the declining substrate concentration as starch is converted to products. Inevitably therefore, the rate of digestion during the early stages of reaction will be faster than the rate at later stages. To define the slower rate as indicative of SDS is therefore in error.

Strong evidence exists that digestion of native granular starch does not follow a single first-order reaction. Instead, the digestion process is best described by two separate first-order reactions that differ in their digestibility rate constant (Butterworth et al., 2012). The reasons for the differences are not fully understood, but starch digested in the faster stage probably represents material that is readily exposed at the surface of granules with easy access to the enzyme. The slower rate is representative of starch within the granule such that slow diffusion of amylase into the granule may become a rate-limiting step (Dhital et al., 2010). Recently we have also shown that encapsulation of starch within plant cell walls (PCW) generates an identifiable slower rate of digestion (Edwards, Warren, Milligan, Butterworth & Ellis, 2014).

Butterworth and co-workers (2012) introduced an improved first-order kinetic model for the analysis of starch hydrolysis using a ‘logarithm of slope’ (LOS) plot. Determination of the slope at several time points of the digestibility curve and plotting the natural logarithm of the slopes against time allows for reliable estimation of *k* and *C*∞ values. The rate constant, *k*, is represented by the negative slope of the rectilinear plot and the total starch digested, *C*∞, can be calculated from the *y*-axis intercept. In addition, the LOS plot approach allows for accurate determination of RDS and SDS starch fractions, if present, from discontinuities in the linear plot. The application of the LOS plot to the data published by Goñi et al. (1997) for the digestion of various cooked food starches revealed a single digestibility constant and therefore demonstrated the questionability of the Englyst classification system. A similar study involved digestion of rice flours prepared under different extrusion conditions (Martínez, Calviño, Rosell, & Gómez, 2014). For both studies, the *k* and *C*∞ values calculated using the LOS plot approach agreed with the first-order kinetic model (Goñi et al., 1997). By and large, however, the number of starches digested and fitted to the LOS plot model has been limited and they have been either in their native or fully gelatinised state.

Upon storage of gelatinised starch, hydrogen bonds begin to form between the polymer chains to provide structural stability. During this phase, the α-glucan chains organise into a tightly packed crystalline structure, which is resistant to α-amylase action. This phenomenon is termed retrogradation (Htoon et al., 2009). Retrogradation is commonly observed in the staling of baked starchy foods such as bread (Hug-Iten, Escher, & Conde-Petit, 2003). Although it is well known that retrograded starch is resistant to amylase action, a lack of kinetic information means that the mechanistic basis for this non-reactivity is not understood. With the widely reported resistance of retrograded starch to amylolysis, it seemed of interest that inclusion of retrograded material in digestibility experiments, analysed using the LOS approach, might reveal useful mechanistic information about the nature of its resistance to amylase.

This paper describes a continuation of our application of first-order kinetics to digestibility plots (Butterworth et al., 2012) to deliver information about the resistance of retrograded starch to α-amylase. Different starches have been digested *in vitro* with α-amylase in their native, gelatinised and 24 h retrograded starch forms. This allows the rate constant, *k*, and *C*∞ to be calculated for the digestibility of native, gelatinised and retrograded starch by amylase. The kinetic work was coupled with spectroscopic studies of starch structure. Fourier transform infrared spectroscopy with attenuated total reflectance (FTIR-ATR) is a surface analytical method that is used to examine the external surface of starch samples. FTIR-ATR therefore allowed determination of the relative proportions of ordered and disordered structures for dispersions of native, gelatinised and 24 h retrograded starch. The FTIR spectra were used to compare the organisation of starch granules with their susceptibility to amylolysis (Sevenou et al., 2002, Warren et al., 2013, Warren et al., 2011).

Given the widely reported interest in retrograded starch, we reasoned that inclusion of retrograded material in digestibility experiments, analysed using the LOS approach, might reveal useful mechanistic information about the nature of the resistance. Taken together with monitoring of starch structural changes we were seeking a better understanding of this interesting starch property of resistance.

## 2 Materials and methods

### 2.1 Starches and chemicals

Wheat starch (Cerestar, CV. GL04) and wild type pea starch were gifts from Prof. C. Hedley and Prof. T. Bogracheva (formerly of the John Innes Centre, Norwich, UK). Purified potato starch was bought from the National Starch and Chemical Company (member of the ICI group, London, UK). Maize, waxy maize and high amylose maize starch were gifts from Dr. C. Pelkman at Ingredion. Rice starch was obtained from Sigma–Aldrich Ltd. Durum wheat grains for durum wheat starch extraction were bought from Millbo, Italy and extracted by the method described in Vansteelandt and Delcour (1999). The method was modified in that the grains were blended using an Ultra-Turrax homogeniser and passed through 250 and 125 μm sieves (Vansteelandt & Delcour, 1999). Phosphate buffered saline (PBS) tablets were purchased from Oxoid Ltd., Basingstoke, Hampshire, UK. When dissolved according to the manufacturer's instructions, a solution of pH 7.3 ± 0.2 at 25 °C is obtained. Porcine pancreatic α-amylase (type 1A, DFP treated) was purchased from Sigma–Aldrich Company Ltd. (Poole, Dorset, UK) as a suspension in 2.9 M NaCl solution containing 3 mM CaCl_2_. The activity stated by the supplier was approximately 1333 units/mg protein. A unit corresponds to 0.97 IU at 25 °C (Tahir, Ellis, & Butterworth, 2010). The purity of α-amylase was verified by SDS-PAGE that also confirmed the molecular weight of 56 kDa. All other reagents were purchased from Sigma–Aldrich Ltd.

### 2.2 Characterisation of starches

Starch moisture was determined gravimetrically by weighing the starch sample onto pre-dried aluminium pans that were then heated overnight at approximately 103 °C. Upon removal, the dried weight was recorded and the moisture content was calculated by the difference between fresh weight and dry weight. The amylose/amylopectin content for the starches was analysed using the iodine dye binding method of Knutson, 1986, Knutson, 2000. Briefly, starch samples including amylose standards, were dissolved in dimethyl sulphoxide (DMSO) containing iodine and water. Samples were then diluted and left for 30 min to allow colour development, before the absorbance was recorded spectrophotometrically at 600 nm (CE 2041, Cecil Instruments, Cambridge, UK). The amylose content was then calculated from appropriate amylose standards. Protein content was determined using the bicinchoninic (BCA) assay, which involves dissolving 50–100 mg of starch in 2% sodium dodecyl sulphate (SDS) before boiling for 2 h to extract the proteins (Baldwin, 2001). Each sample was then centrifuged at 13,000 rpm for 10 min before 50 μL was removed and tested by the BCA assay.

### 2.3 Preparation and digestion of starches

A 5 mg/mL solution of native starch was prepared in PBS and incubated at 37 °C with 4.5 nM (approximately 0.33 IU) porcine pancreatic α-amylase on an inclined rotating table to allow constant end-over-end mixing. Samples were withdrawn at timed intervals up to 120 min and transferred to ice-cold 0.3 M Na_2_CO_3_ stop solution in Eppendorf tubes. The ice-cold stop solution rapidly cools the reaction sample and raises the pH to a level where amylase is no longer active. The Eppendorfs were then centrifuged and the supernatant was collected and used for determination of reducing sugar concentration by a Prussian blue assay (Slaughter, Ellis, & Butterworth, 2001). Each sample was diluted in water before 150 μL aliquots of solution A (16 mM KCN, 0.19 M Na_2_CO_3_ in distilled H_2_O) and solution B (1.18 mM K_3_Fe(CN)_6_ in distilled H_2_O) were added. Samples were then boiled for 15 min and allowed to cool at room temperature before 750 μL of solution C (3.11 mM NH_4_Fe(SO_4_)_2_, 0.1% (w/w) SDS, 0.2% (v/v) H_2_SO_4_ in distilled H_2_O) was added. The tubes were allowed to stand at room temperature for 2.5 h before the absorbance was recorded at 695 nm. Maltose standards ranging from 0 to 100 μM were treated similarly and used for quantification of the content of reducing sugar expressed as maltose equivalents. For studies of gelatinised material, starch was heated for 20 min at 90 °C and then cooled to 37 °C before α-amylase was added to produce a final concentration of 2.25 nM. Starch containing retrograded material was prepared by similar heat treatment followed by a storage period of 24 h at room temperature before α-amylase was added to give an enzyme concentration of 2.25 nM for digestibility measurements.

The slopes of digestibility curves were measured at various time points throughout the incubation period, converted to logarithmic form and then fitted to the first-order kinetic model (see Butterworth et al. (2012) for details of the relatively straightforward calculations). Accurate estimate of the pseudo rate constant, *k*, and the total digestible starch, *C*∞, are obtainable from plots of LOS against time.

### 2.4 FTIR-ATR spectroscopy

Absorbance spectra were recorded using a Perkin Elmer Spectrum Two^®^ FTIR spectroscope equipped with a SensIR technologies IR II Durascope^®^ diamond cell ATR device. The spectroscope was fitted with PerkinElmer Spectrum 10^©^ software for peak detection. This was accompanied with a diamond crystal with an angle of incidence of 45°. A 10 μL aliquot of 10 mg/mL starch in distilled H_2_O was placed on the surface crystal of the ATR device. The starch sample was then scanned over a wavelength range of 4000 cm^−1^ to 550 cm^−1^, and averaged from a total of 24 scans with a resolution of 4 cm^−1^. For gelatinised material, a 10 mg/mL mixture was heated in distilled H_2_O at 90 °C for 20 min, returned to room temperature (20 °C) and allowed to cool to 37 °C, before FTIR spectra were taken. Retrograded starch was prepared by a similar heat treatment procedure but stored at room temperature for 24 h before a spectrum was taken. A spectrum for distilled H_2_O was also measured and subtracted from the final sample spectra before the data were normalised and compared (Warren et al., 2011).

## 3 Results and discussion

### 3.1 Starch characterisation

Starches vary in their physiochemical properties and granular structure and therefore characterisation is important when comparisons of digestibility are to be made. All starches used in this investigation were tested for their protein, amylose and moisture content and the results are shown in Table 1. The protein content for all the starches ranged between 0.05 and 0.46%. The percentage of amylose varied depending upon the starch source with waxy maize having the lowest amylose content of 1% and high amylose maize having the highest with 79%. The moisture content values of the starch samples varied between 11 and 16%.

**Table 1:** Characteristic of starches used in this investigation. The protein and amylose values in this table are presented on a dry weight basis with mean values ± standard error of the mean (s.e.m.) from three to four replicates.

Starch	Protein (%)	Amylose (%)	Moisture (%)
Wheat	0.14 ± 0.01	20.3 ± 0.9	11.1 ± 0.6
Potato	0.05 ± 0.00	15.5 ± 1.9	16.3 ± 0.5
Durum wheat	0.10 ± 0.00	29.2 ± 1.5	15.1 ± 0.4
Wild type pea	0.25 ± 0.01	26.8 ± 1.6	12.8 ± 1.9
Rice	0.20 ± 0.03	17.8 ± 1.1	15.0 ± 0.6
Maize	0.16 ± 0.03	22.8 ± 0.8	11.3 ± 0.6
Waxy maize	0.32 ± 0.04	1.2 ± 0.1	13.7 ± 0.4
High amylose maize	0.46 ± 0.02	79.1 ± 4.3	12.0 ± 1.2

### 3.2 FTIR-ATR

**Fig. 1:**
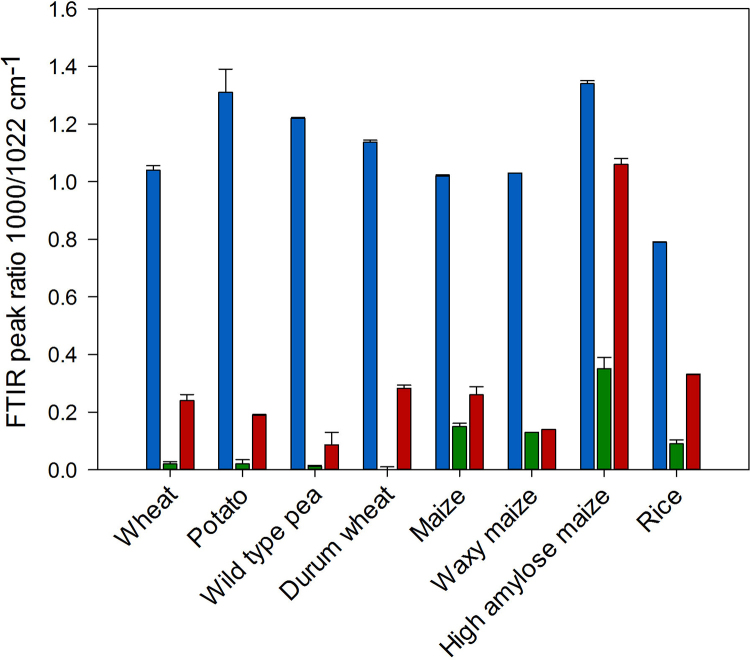
FTIR-ATR 1000/1022 cm^−1^ peak ratio of native (blue), gelatinised (green) and 24 h retrograded (red) starches. All values are presented as mean values ± standard error of the mean (s.e.m.) from three to four replicates. (For interpretation of the references to color in this figure legend, the reader is referred to the web version of this article.)

From previous work it has been established that characteristic peaks at 1000 cm^−1^ and 1022 cm^−1^ are associated with the maximum absorbance when examining the surface of starch granules. The 1000 cm^−1^ peak is characteristic of the ordered regions and the 1022 cm^−1^ peak is associated with the amorphous regions on the starch surface (Capron et al., 2007, Sevenou et al., 2002, Warren et al., 2011). The peak ratio of 1000/1022 cm^−1^ can therefore be used as an estimate of the degree of ordered to disordered α-glucan chains at the granule surface. Fig. 1 shows the peak ratio between 1000 cm^−1^ and 1022 cm^−1^ for native, gelatinised and 24 h retrograded starch. Native starch samples have a relatively high peak ratio indicating that starch at the granule surface is mainly in an ordered state. The peak ratio decreases dramatically upon gelatinisation due to a loss in crystallinity; however for starch containing retrograded material, the ratio begins to increase, which is evidence of recrystallised α-glucan chains. This is expected to be mainly recrystallised amylose as amylopectin takes several days to recrystallise (Chung et al., 2006, Cui and Oates, 1997, Sasaki et al., 2000). This explains the high degree of order observed in high amylose maize starch stored for 24 h compared with waxy maize starch, which shows no change (Fig. 1).

### 3.3 *In vitro* starch digestibility

**Fig. 2:**
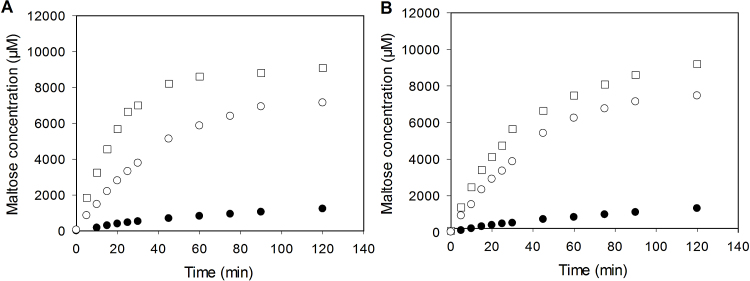
Digestibility curves of native (●), gelatinised (□) and 24 h retrograded (○) starches. (A) Wheat starch and (B) wild type pea starch.

**Fig. 3:**
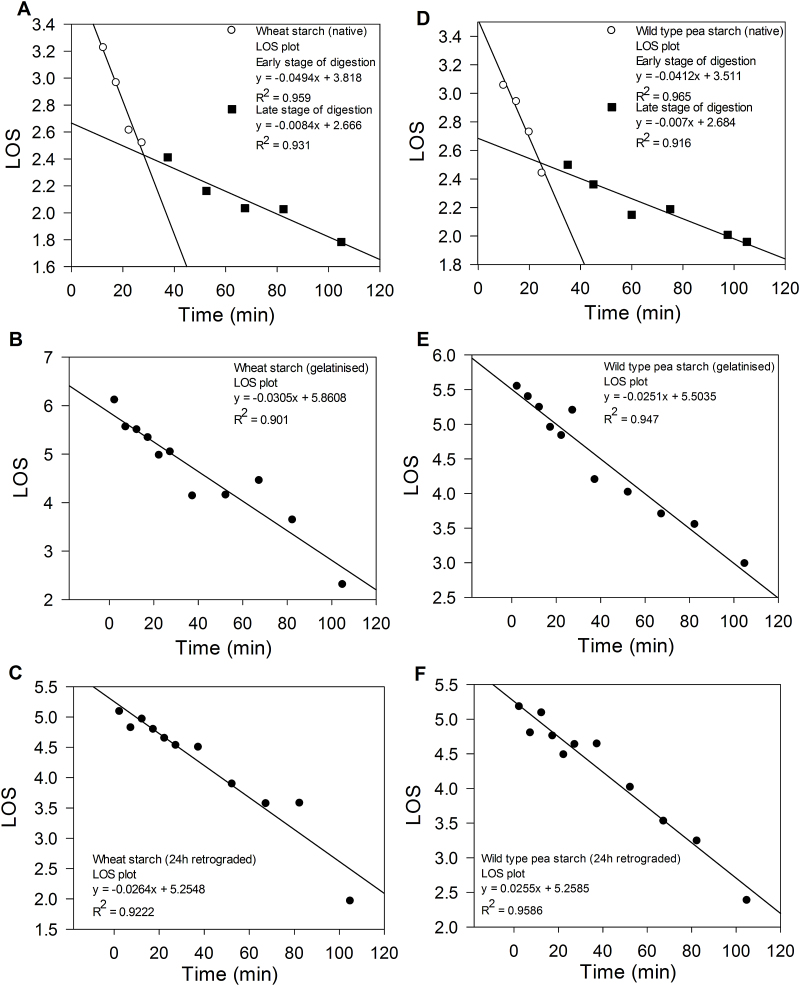
LOS plot of native (A), gelatinised (B) and 24 h retrograded (C) wheat starch digestion; LOS plot of native (D), gelatinised (E) and 24 h retrograded (F) wild type pea starch digestion. Data for native and gelatinised wheat and wild type pea digestion are reproduced from Butterworth (2012). All LOS plots were obtained from three to four replicate digestion assays.

Native starches were digested with 4.5 nM α-amylase but gelatinised and 24 h retrograded starches were digested with 2.25 nM α-amylase. A 4.5 nM α-amylase concentration was used for native starches because of the relatively slow starch digestion rate. Increasing the concentration of amylase to increase the reaction rate improved the precision of rate measurements. It is important therefore to note that the values for the pseudo-first-order rate constants calculated for native starches have to be halved before comparison with the rate constants for gelatinised and 24 h retrograded starches because of the difference in amylase concentration. The digestibility curves and LOS plots for the digestion of native, gelatinised and 24 h retrograded wheat and wild type pea starches are shown in Fig. 2, Fig. 3. Using the LOS plots, *k* and *C*∞ values were calculated for the digestion of all starches in their native, gelatinised and 24 h retrograded forms (Table 2).

**Table 2:** Rate constant (*k*) and percentage of total starch digested after 2 h incubation (*C*∞) calculated from the LOS plots for native, gelatinised and 24 h retrograded starches. The *C*∞ percentages are relative to the dry weight of starch included in reaction mixtures.

Starch	Native				Gelatinised		24 h Retrograded	
	Rapid		Slow		*k* (min^−1^)	*C*∞ (%)	*k* (min^−1^)	*C*∞ (%)
	*k* (min^−1^)	*C*∞ (%)	*k* (min^−1^)	*C*∞ (%)				
Wheat	0.049	7.0	0.008	13.1	0.040	70.8	0.030	55.8
Potato	0.040	1.0	0.006	1.6	0.028	86.8	0.025	70.0
Wild type pea	0.041	6.3	0.007	15.6	0.025	77.0	0.026	62.5
Durum wheat	0.055	6.5	0.006	16.3	0.012	83.3	0.011	62.5
Rice	0.060	10.1	0.009	26.8	0.021	76.0	0.024	59.0
Maize	0.035	5.4	0.006	17.4	0.022	75.4	0.022	57.8
Waxy maize	0.070	11.7	0.012	15.1	0.043	77.4	0.044	73.0
High amylose maize	0.042	1.9	0.007	3.6	0.024	78.1	0.027	55.1

The wheat and wild type pea digestion plots shown in Fig. 2, Fig. 3 are taken from Butterworth et al. (2012), with the addition of the LOS plot for 24 h retrograded wheat and wild type pea starch. The LOS plots for durum wheat, potato, maize and rice starch show similar digestion profiles and the calculated *k* and *C*∞ are shown in Table 2. The moisture content was taken into account when calculating *C*∞ for all starches. This is the likely explanation of why the *C*∞ values reported here for wheat and wild type pea starch are higher than those previously published in Butterworth et al. (2012), where moisture content was not taken into account in estimations of the weight of starch present in the reaction mixture.

Native starches are known to be much more resistant to digestion as the result of a high degree of crystallinity in the starch structure with α-glucan chains being tightly packed. This packing of α-glucan chains supposedly limits the ability of glucan residues to form hydrogen bonds with specific amino acid side chains within the α-amylase active site so that catalytic activity is impaired (Imberty, Chanzy, & Perez, 1988).

**Fig. 4:**
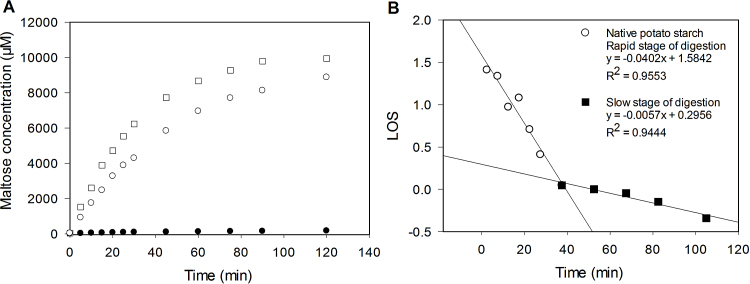
(A) Digestibility curves of native (●), gelatinised (□) and 24 h retrograded (○) potato starch digestion; (B) LOS plot of native potato starch digestion at 37 °C with 4.5 nM porcine pancreatic α-amylase.

The LOS plots revealed a discontinuity that occurred between 20 and 30 min of digestion time suggesting that native starches are digested in two separates phases. The more rapid phase we designate as readily accessible starch and the slower phase represents less accessible α-glucan chains (Butterworth et al., 2012). However, the native potato LOS plot revealed that the more rapid phase was of relatively long duration in that it lasted for up to 40 min (Fig. 4). This may suggest substrate availability at the surface of potato starch is particularly limited since the slow rate of catalysis in the first phase (relative to the other starches (Table 2)) can be explained by a reaction taking place at a low substrate concentration. Also, it needs to be noted that the large size of granules of potato starch means that the relative surface area of available substrate is small and therefore the binding of amylase is less favourable (Warren et al., 2011). This is likely to have a noticeable impact on the rate of reaction. Therefore the digestion of the limited α-glucan chains represented by the initial ‘rapid’ phase is inevitably extended. The reaction characterised by the slower phase represents hydrolysis of less-available starch and is reflected in the low *k* and *C*∞ values (see Table 2).

Upon heating at 90 °C, the granule integrity and degree of crystallinity becomes disrupted and the granular starch becomes disordered. The α-glucan chains become more exposed to the solvent and thus the susceptibility for attack by α-amylase is increased. Therefore the number of α-glucan chains which can interact favourably with α-amylase is increased, *i.e.*, there is an increase in the concentration of available substrate (Butterworth et al., 2011). The rapid phase is characterised by a higher *k* value, because the exposed α-glucan chains on the granule surface are readily available for attack by α-amylase. The enzyme will catalyse a reaction at a rate commensurate with its inherent turnover number (Butterworth et al., 2011).

At the enzyme concentration used in our assays, the more rapid phase lasts for approximately 30 min, which is then succeeded by the slower phase. The low *k* value for the slow phase can be explained by the greater difficulty that α-amylase experiences in binding to α-glucan chains buried within the starch granule, and/or the slow rate of diffusion of amylase through the granule to reach susceptible glucan chains (Dhital et al., 2010, Zhang et al., 2006). After gelatinisation, α-glucan chains are more accessible and are therefore directly available for amylase action. As a result, not only is the digestibility rate constant raised, but *C*∞ also increases by approximately 10-fold.

During storage for 24 h, starch begins to retrograde and slowly recrystallise. The increased crystallinity is expected to result in fewer available α-glucan chains to which α-amylase can bind and thus reduce the susceptibility of retrograded starch to digestion (Htoon et al., 2009, Hug-Iten et al., 2003, Liu et al., 2007).

Retrograded starch is primarily retrograded amylose because the relatively short and mostly linear amylose chains can re-associate within 48 h, whereas the bulky amylopectin can take several days to re-associate (Khanna and Tester, 2006, Sajilata et al., 2006). Sievert and workers have shown the development of crystalline starch, using differential scanning calorimetry (DSC) and X-ray diffraction (XRD), for autoclaved-cooled amylomaize starch, a process which accelerates the amount of retrograded starch (Sievert et al., 1991, Sievert and Pomeranz, 1989, Sievert and Pomeranz, 1990). In accordance with the increased crystallinity, the experimentally determined *C*∞ value for retrograded high amylose maize starch was seen to decrease, whereas an almost negligible change in *C*∞ was observed with retrograded waxy maize (*i.e.*, high amylopectin) starch. The absence of discontinuities in LOS plots obtained for gelatinised and 24 h retrograded starches, suggests that retrogradation has not brought about the production of a new structural element that can be digested, albeit at a slower rate.

In our preparation of retrograded starch, the bulk of the starch material present is still in the gelatinised, rapidly digested, form but the quantity of non-digestible starch is increased by retrogradation of amylose particularly (Fredriksson et al., 1998, Tester et al., 2004). There is no direct evidence to suggest that retrograded material *per se* is digested at a slower rate than gelatinised starch since *k* values for gelatinised and 24 h retrograded starches are similar. If retrograded starch is virtually inert towards α-amylase, the measured *k* value will only reflect the digestion of starch in the digestible/accessible form that remains within the preparation, *i.e.*, material that has not become retrograded. Therefore, the decrease in *C*∞ values, coupled with unchanged *k* values, for the retrograded preparations relative to the gelatinised starch, seems to provide evidence that retrograded starch is inert to α-amylase action over the time scale of the experiment. Inhibition by product maltose of amylase activity seems an unlikely explanation for the decrease in *C*∞. The *K*_*i*_ for maltose at 9–14 mM is indicative of a weak inhibitor (Seigner et al., 1987, Warren et al., 2012) and its effects would therefore only become detectable at very low starch concentrations coupled with high accumulation of high maltose. Since the digestibility constant found for the retrograded samples is identical with that for gelatinised (non-retrograded starch), it may be concluded that retrograded starch does not have a direct inhibitory action on amylase.

## 4 Conclusions

*In vitro* digestibility curves for different botanical starches in their native, gelatinised and retrograded starch forms are well fitted by the first-order kinetic equation. This allows for the accurate determination of *C*∞ and *k* values from a LOS plot. All starches in their native or processed forms display different digestibility rates due to variations in the proportion of amorphous material, resulting in a distinctive rate constant for different starch fractions. The digestibility rate constants of the various gelatinised starches are virtually identical because they represent an intrinsic catalytic property of amylase (Butterworth et al., 2012). Where distinct rapid and slower phases can be identified, the LOS method also allows quantification of the relative amounts of readily available starch fractions.

Upon 24 h retrogradation, the quantity of digestible starch is decreased compared with gelatinised starch due to changes in starch crystallinity/order. Evidence for changes in starch crystallinity was obtained from FTIR-ATR spectra, which indicated an increase in the degree of ordered structure.

This study illustrates that LOS plots can be applied to different botanical sources of starches and that the rate and extent of digestion can be accurately determined for native and processed starches. Values for the maximum extent of digestion are important for predicting the total digestibility *in vivo* of starch in foods and the consequent glycaemia.
